# A patient with Alagille syndrome had a novel JAG1 gene mutation

**DOI:** 10.1016/j.gendis.2025.101759

**Published:** 2025-07-03

**Authors:** Ziting Peng, Yao Chen, Li Zhang, Tongdong Shi, Na Wang

**Affiliations:** Department of Infectious Diseases, Key Laboratory of Molecular Biology for Infectious Diseases (Ministry of Education), Institute for Viral Hepatitis, The Second Affiliated Hospital, Chongqing Medical University, Chongqing 400065, China

Alagille syndrome (ALGS) is a rare, autosomal dominant disorder that affects multiple systems. It was first reported by Alagille et al in 1969 and further elaborated on its clinical, biochemical, and histological features in 1975. The main organs involved in the disease include the liver, heart, eyes, bones, and kidneys. As a rare genetic disease, ALGS is primarily observed in children, and confirmed cases in adults are rarely reported. Therefore, we report a case of an adult female who has been diagnosed with ALGS, and the genetic testing revealed a heterozygous mutation at position 933_934 of the Jagged 1 (JAG1) gene, which was discovered for the first time.

We report a case involving a 28-year-old female patient from Yunnan Province, China. In May 2023, abnormal liver function was detected in the patient during the physical examination in a hospital: alanine aminotransferase (ALT): 63.0 U/L; alkaline phosphatase (ALP): 161.1 U/L; gamma-glutamyl transferase (GGT): 722.0 U/L; total bile acid (TBA): 33.65 μmol/L. The patient continued to improve the upper abdominal magnetic resonance imaging (MRI) enhancement and magnetic resonance cholangiopancreatography (MRCP) examination, and the results revealed hepatomegaly, with notable swelling observed in the caudate lobe and left inner lobe of the liver. Additionally, diffuse lesions were observed throughout the liver. The patient's other tests for alpha-fetoprotein (AFP), hepatitis B surface antigen (HBsAg), anti-hepatitis A virus (anti-HAV) IgM, anti-hepatitis C virus (anti-HCV) IgM, anti-hepatitis E virus (anti-HEV) IgM, immunoglobulins IgA, IgM, IgG, and IgE, complement levels, and antinuclear antibodies (ANA) spectrum all yielded negative results. The patient came to our hospital on July 17, 2023.

After being admitted to our hospital, the patient's liver function was re-examined to establish baseline values: ALT: 47.0 U/L; aspartate transferase (AST): 62.0 U/L; ALP: 203.0 U/L; GGT: 1132.0 U/L; total bilirubin (T-Bil): 12.7 μmol/L; direct bilirubin (D-Bil): 5.3 μmol/L; TBA: 9.2 μmol/L. A treatment regimen aimed at protecting the liver and reducing elevated liver enzyme levels was promptly initiated for the patient. We conducted a follow-up evaluation of her liver function test 1 week later: ALT: 50.0 U/L; AST: 33.0 U/L; ALP: 131.0 U/L; GGT: 653.0 U/L; T-Bil: 11.3 μmol/L; D-Bil: 5.7 μmol/L; TBA: 18.7 μmol/L. The results were better than before. To identify the underlying cause of the abnormal liver function, we conducted a comprehensive physical examination on the female patient and collected her detailed medical history. We found no anomaly on physical examination, and the patient denied any history of medication use, alcohol consumption, or other factors that could lead to liver damage. It was noteworthy that we found that the patient exhibited distinctive physical characteristics, including a small stature, blindness in the right eye, a prominent forehead, and widely spaced eyes, resulting in a unique facial appearance. After obtaining a detailed medical history, it was discovered that she had congenital blindness in the right eye and had experienced repeated episodes of abnormal liver function during infancy. But the cause was not clear. Once she had been infected with hepatitis B virus and received interferon treatment for 1 month. Therefore, considering the patient's medical history and distinctive physical features, we suspect that the patient may be suffering from an inherited metabolic liver disease, most likely ALGS. So, follow-up examinations, especially liver tissue biopsy and genetic testing, were conducted to further investigation.

No obvious signs of ALGS were found on ophthalmic examination, echocardiography, and anterolateral X-ray films of the vertebral body. The pathologic results of the liver biopsy showed scattered focal necrosis in the hepatic lobules, feathery degeneration of hepatocytes with mild cholestasis, capillary bile duct dilation, and mild portal inflammation. From immunohistopathology results of CK19, we found poorly developed small bile ducts in individual portal areas (Fig. 1A). We conducted genetic testing on this family, and found c.933-934dup mutation in exon in the proband (p. Cys312PhefsTer101), absent in the unaffected parents. This suggests that the mutation of this gene is spontaneous (Fig. 1B, C). The patient's ALGS was ultimately confirmed through genetic testing, and it was discovered that the gene mutation occurred at a novel site: nucleotide repeats at 933 and 934 on the JAG1 gene.

**Figure 1:**
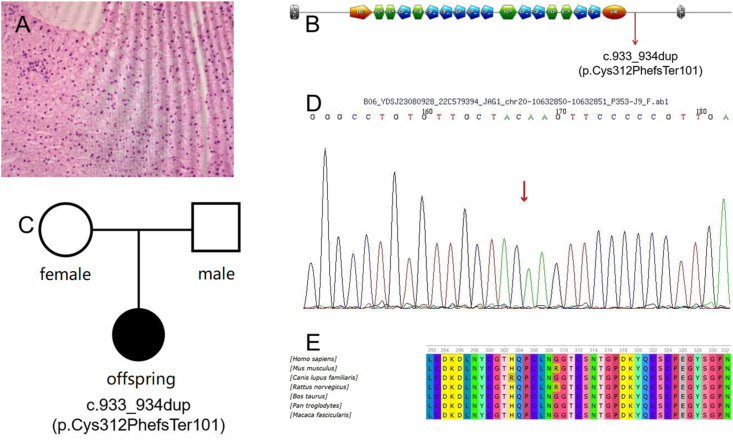
Pathological image of liver puncture. **(A)** The image shows focal necrosis in the hepatic lobules, feathery degeneration of hepatocytes with mild cholestasis, capillary bile duct dilatation, and poorly developed small bile ducts in individual portal areas. **(B)** The prediction diagram of JAG gene secondary structure. **(C)** The pedigrees of the family of three. **(D)** Heterozygous missense mutations in the JAG1 gene, c.933-934dup. **(E)** The amino acids at position 312 are highly conserved across various species.

ALGS is a rare autosomal dominant genetic disorder caused by mutations in one of two genes, JAG1 and NOTCH2, which are essential components of the Notch signaling pathway. The JAG1 gene is located on chromosome 20, while the NOTCH2 gene is located on chromosome 1.^1^ Currently, most of the reported patients have pathogenic mutations of JAG1, and a small number have NOTCH2 mutations. The disease is frequently diagnosed in infants and young children presenting with cholestasis. ALGS patients are susceptible to involvement of multiple organs including the liver, heart, kidneys, bones, and eyes, which can be mainly manifested as cholestasis (pruritus, Jaundice), heart disease (abnormalities in the right ventricular outflow tract, peripheral pulmonary artery stenosis), butterfly vertebrae, eye abnormalities, and distinctive facial features. Different age stages are mainly associated with different clinical manifestations, and there can be significant individual differences in these presentations.

The pathologic findings of ALGS were primarily focused on biliary duct scarcity. However, there are only a few reported cases worldwide of adults meeting the traditional diagnostic criteria for ALGS. In these cases, there may be no bile duct deficiency observed in some patients.^2^ Therefore, we think that traditional diagnostic criteria are not applicable to adult ALGS and the diagnosis should rely more on genetic testing than clinical features.

ALGS has features that are not completely explicit. The mutant gene does not have a specific phenotype, and there is no obvious relationship with the severity of the phenotype. Consequently, up to 96% of patients with ALGS are diagnosed through genetic testing.^3^ Among patients diagnosed with ALGS, 94%–96% have JAG1 gene mutations, mainly involving nucleotide deletion, repetition, nonsense mutation, and insertion. Only 2%–3% of patients have NOTCH2 mutations, and most of these mutations are missense mutations.^3^^,^^4^ We found a novel mutation of JAG1 gene in the patient's test report: c.933_934dup (Fig. 1D). It refers to the two base repeats at positions 933 to 934 on the JAG1 gene, which affects the splicing process. This mutation can lead to a frame shift mutation in the amino acid sequence encoded by the JAG1 gene (position 312 mutated from cysteine to phenylalanine), and produce abnormal protein products. This mutation has not been reported in other ALGS patients to date. Therefore, we speculate that c.933_934dup may be a pathogenic mutation of ALGS. What's more, this variant was exclusive to this patient with ALGS but absent in her parents. This suggests that the mutation in the patient's gene occurred spontaneously, and it is rare. This suggests variable disease penetrance, and we suspect the potential influence of environmental factors in conjunction with the JAG1 mutation to manifest the phenotype. Second, comparative genomic analysis revealed evolutionary conservation of cystine at position 312 across multiple animal species, suggesting having important biological functions (Fig. 1E). JAG1 gene mutations have a high *de novo* mutation rate. It has been reported that approximately 60% of patients with JAG1 gene mutations do not have those mutations present in both parents.^5^ So, in addition to the known JAG1 mutation, there are other genomic modifications that may cause the disease, which need to be proved by further studies.

In this work, we report a novel mutation site in a patient with ALGS, which has arisen spontaneously rather than being inherited from the patient's parents, and we speculate that c.933_934dup may be the cause of this patient's disease. The diagnosis of ALGS can be challenging due to the lack of a clear genotypic–phenotypic correlation. Furthermore, the causes of bile duct deficiency and cholestasis in ALGS are complex and varied, increasing the diagnostic complexity.

## CRediT authorship contribution statement

**Ziting Peng:** Writing – review & editing, Investigation, Conceptualization, Writing – original draft, Data curation. **Yao Chen:** Writing – review & editing, Data curation, Investigation, Conceptualization. **Li Zhang:** Data curation, Writing – review & editing, Conceptualization. **Tongdong Shi:** Supervision, Conceptualization, Writing – review & editing, Funding acquisition. **Na Wang:** Visualization, Conceptualization, Writing – review & editing, Funding acquisition.

## Ethics declaration

This project has been approved by the Ethics Committee of the Second Affiliated Hospital of Chongqing Medical University (Approval number: 7). The patient provided informed consent for publication of the case.

## Funding

This work was supported by the First Batch of Key Disciplines on Public Health in Chongqing, Health Commission of Chongqing, China, and Medical Scientific Research Foundation of Chongqing of China (2022MSXM048).

## Conflict of interests

The authors declared no conflict of interests.
